# Benefits of Qigong as an integrative and complementary practice for health: a systematic review*

**DOI:** 10.1590/1518-8345.3718.3317

**Published:** 2020-07-15

**Authors:** Bruna Francielle Toneti, Rafael Fernando Mendes Barbosa, Leandro Yukio Mano, Luana Okino Sawada, Igor Goulart de Oliveira, Namie Okino Sawada

**Affiliations:** 1 Universidade de São Paulo, Escola de Enfermagem de Ribeirão Preto, PAHO/WHO Collaborating Centre at the Nursing Research Development, Ribeirão Preto, SP, Brazil.; 2 Scholarship holder at the Coordenação de Aperfeiçoamento de Pessoal de Nível Superior (CAPES), Brazil.; 3 Universidade de São Paulo, Instituto de Ciências Matemáticas e de Computação, São Carlos, SP, Brazil.; 4 Scholarship holder at the Fundação de Amparo à Pesquisa do Estado de São Paulo (FAPESP), Brazil.; 5 School of Computing and Information Science, Florida International University, Miami, FL, United States of America.; 6 Universidade Estadual do Norte do Paraná, Centro de Ciências Tecnológicas, Bandeirantes, PR, Brazil.; 7 Universidade Federal de Alfenas, Escola de Enfermagem, Alfenas, MG, Brazil.

**Keywords:** Complementary Therapies, Medicine, Chinese Traditional, Qigong, Systematic Review, Evidence-Based Practice, Nursing, Terapias Complementares, Medicina Tradicional Chinesa, Qigong, Revisão Sistemática, Prática Clínica Baseada em Evidências, Enfermagem, Terapias Complementarias, Medicina China Tradicional, Qigong, Revisión Sistemática, Práctica Clínica Basada en la Evidencia, Enfermería

## Abstract

**Objective:**

to analyze, in the literature, evidence about the benefits of the integrative and complementary practice of *Qigong* with regard to the health of adults and the elderly.

**Method:**

a systematic review by searching for studies in the PubMed, CINAHL, LILACS, EMBASE and Cochrane Library databases. Randomized and non-randomized clinical trials were included; in Portuguese, English and Spanish; from 2008 to 2018. The Preferred Reporting Items for Systematic Reviews and Meta-Analyses strategy was adopted, as well as the recommendation of the Cochrane Collaboration for assessing the risk of bias in the clinical trials analyzed.

**Results:**

28 studies were selected that indicated the benefit of the practice to the target audience, which can be used for numerous health conditions, such as: cancer; fibromyalgia; Parkinson’s disease; Chronic Obstructive Pulmonary Disease; Burnout; stress; social isolation; chronic low back pain; cervical pain; buzz; osteoarthritis; fatigue; depression; and cardiovascular diseases. However, there was a great risk of bias in terms of the blinding of the research studies.

**Conclusion:**

the practice of *Qigong* produces positive results on health, mainly in the medium and long term. This study contributes to the advancement in the use of integrative and complementary practices in nursing, since it brings together the scientific production in the area from the best research results available.

## Abstract (pt)

**Objetivo:**

analisar na literatura as evidências sobre os benefícios da prática integrativa e complementar de *Qigong* à saúde de pessoas adultas e idosas.

**Método:**

revisão sistemática mediante a busca de estudos nas bases de dados PubMed, CINAHL, LILACS, EMBASE e *Cochrane Library*. Foram incluídos ensaios clínicos randomizados e não randomizados; em português, inglês e espanhol; de 2008 a 2018. Adotou-se a estratégia *Preferred Reporting Items for Systematic Reviews and Meta-Analyses*, bem como a recomendação da Colaboração *Cochrane* para a avaliação do risco de viés dos ensaios clínicos analisados.

**Resultados:**

foram selecionados 28 estudos que indicaram o benefício da prática para o público-alvo, podendo ser utilizada para inúmeras condições de saúde, como: câncer; fibromialgia; Doença de *Parkinson*; Doença Pulmonar Obstrutiva Crônica; *Burnout*; estresse; isolamento social; lombalgia crônica; dor cervical; zumbido; osteoartrite; fadiga; depressão; e doenças cardiovasculares. Notou-se, contudo, grande risco de viés no que tange ao cegamento das pesquisas.

**Conclusão:**

a prática de *Qigong* produz resultados positivos sobre a saúde, principalmente, a médio e longo prazo. Este estudo contribui para o avanço no uso de práticas integrativas e complementares na enfermagem uma vez que reúne a produção científica na área a partir dos melhores resultados de pesquisas disponíveis.

## Abstract (es)

**Objetivo:**

analizar las evidencias presentes en la literatura acerca de los beneficios del *Qigong* como práctica integradora y complementaria en relación a la salud de adultos y ancianos.

**Método:**

revisión sistemática mediante la búsqueda de estudios en las siguientes bases de datos: PubMed, CINAHL, LILACS, EMBASE y *Cochrane Library*. Se incluyeron ensayos clínicos aleatorizados y no aleatorizados en portugués, inglés y español, de 2008 a 2018. Se adoptó la estrategia *Preferred Reporting Items for Systematic Reviews and Meta-Analyses*, así como la recomendación de la Colaboración *Cochrane* empleada para evaluar el riesgo de sesgo de los ensayos clínicos analizados.

**Resultados:**

se seleccionaron 28 estudios que indicaron los beneficios de la práctica para el público objetivo, empleada en la afección de numerosos problemas de salud como cáncer, fibromialgia, Mal de *Parkinson*, Enfermedad Pulmonar Obstructiva Crónica, *Burnout*, estrés, aislamiento social, lumbalgia crónica, dolor cervical, tinnitus, osteoartritis, fatiga, depresión y enfermedades cardiovasculares. No obstante, se registró un gran riesgo de sesgo en lo que se refiere al procedimiento de enmascaramiento de las investigaciones.

**Conclusión:**

practicar *Qigong* produce resultados positivos en la salud, principalmente, a mediano y largo plazo. Este estudio contribuye al avance en el uso de prácticas integradoras y complementarias en enfermería, ya que reúne la producción científica en el área, seleccionada a partir de los mejores resultados de las investigaciones disponibles.

## Introduction

Also known as alternative and/or complementary practices ( *Práticas Integrativas e Complementares*, PICs), integrative therapies are aimed at achieving physical and mental well-being and are able to alleviate symptoms arising from health conditions and conventional treatments^(^ 1 ^-^ 2 ^)^.

In 2006, in Brazil, PICs were recognized, following the publication of the National Policy on Integrative and Complementary Practices ( *Política Nacional de Práticas Integrativas e Complementares*, PNPIC), by Ordinance No. 971, including the following in the Unified Health System ( *Sistema* Único *de Saúde*, SUS): Traditional Chinese Medicine (TCM)/Acupuncture; Homeopathy; Medicinal Plants, Phytotherapy; Anthroposophical Medicine; and Crenotherapy-Social Thermalism. Eleven years later, with Ordinance No. 849, others were added: Art Therapy, Ayurveda, Biodance, Circular Dance, Meditation, Music Therapy, Naturopathy, Osteopathy, Chiropractic, Reflexotherapy, Reiki, Shantala, Integrative Community Therapy, and Yoga^(^ 3 ^-^ 4 ^)^.

Recently, in 2018, by Ordinance No. 702, the following were added to the system: Aromatherapy, Apitherapy, Bioenergetics, Family Constellation, Chromotherapy, Geotherapy, Hypnotherapy, Laying on of Hands, Ozone Therapy, and Flower Therapy^(^ 5 ^)^. The fact that PICs have their demand increased, as well as their insertion in the SUS, points to the beginning of a movement in Brazil in the search for qualifying the form of health care offered to the population, which is gradually more comprehensive, resolutive, and universal^(^ 1 ^)^.

*Qigong* is a TCM PIC that meets this perspective of health care. And, as already discussed, it is a practice with a high level of recognition for its positive health results. During the process, there is an improvement in the transport of energy and blood through the established body-mind relationship, which influences the blood, the essence, body fluids, and the mind, essential to the human being. In this way, it is possible to adjust and harmonize the flows of *Qi* and the *Yin-Yang* of the body, therefore promoting health^(^ 6 ^)^.

A study in the area has progressively explored the results of this therapy in different target audiences, for example, people with advanced age and others with chronic health conditions that are not susceptible to transmission, in order to understand its influence in terms of health^(^ 6 ^)^. However, more evaluations, from studies with recognized methods, are necessary with regard to the physiology involved in the results of therapy, so that it is possible to develop what is known in the area regarding its relationship with the health of practitioners, a reason that encouraged the development of the present study.

Practices that develop both physical and mental conditions and Quality of Life (QoL), such as the PICs, are the foundation for the search for quality nursing care, carried out during the sharing of knowledge between professionals and users of health. In this sense, there is an appreciation of their active participation in this process^(^ 2 ^)^.

The fact that nurses use PICs is related to a humanizing movement for the integration of care, which covers its dissemination, as well as the act of making them legitimate in this context. Although the literature presents solid evidence, there is little use of these practices by professionals in the area and patients^(^ 1 ^-^ 2 ^)^.

Therefore, in order to build a scientific contribution, in addition to optimizing the action of nursing in both care and health production, research that explores the results of the PICs, such as *Qigong*, with a focus on those related to nursing, are essential. That said, the objective of this systematic review was to analyze, in the literature, evidence about the benefits of the integrative and complementary practice of *Qigong* with regard to the health of adults and the elderly.

## Method

The literature Systematic Review (SR) method makes it possible to gather, analyze, and synthesize scientific productions regarding a certain clinical issue, so that it is possible to understand, discuss, and establish clinical actions based on evidence^(^ 7 ^)^.

The methodological trajectory followed was based on the Preferred Reporting Items for Systematic Reviews and Meta-Analyses (PRISMA), through its checklist of items and flow chart for the development of a SR^(^ 8 ^-^ 9 ^)^.

In order to construct the research question of this SR, the PICO strategy was used: Patient/Population/Problem of interest (P): adults and the elderly; Intervention/Area of Interest (I): practice of *Qigong*; Comparison (C): not applicable; and Outcomes/Results (O): benefits of practicing *Qigong* to health. With it, it is possible to identify keywords on the subject. The strategy is essential with regard to the development of the search strategy for relevant primary studies in databases^(^ 9 ^)^.

Thus, the following question was obtained: Does the practice of *Qigong* have beneficial effects on the health of adults and the elderly? The research of the primary studies, carried out from the descriptors presented below, took place in the following databases: *N* ational Library of Medicine National Institutes of Health (PubMed), Cumulative Index to Nursing and Allied Health Literature (CINAHL); Latin American and Caribbean Health Sciences Literature ( *Literatura Latino-Americana e do Caribe em Ciências da Saúde*, LILACS); Excerpta Medica Database (EMBASE); and Cochrane Controlled Register of Trials (CENTRAL) - *Cochrane Library*.

For that, descriptors were used, as well as synonyms, in agreement with the Health Sciences Descriptors ( *Descritores em Ciências da Saúde*, DeCS), the Medical Subject Headings (MeSH) and the Emtree from Elsevier Life Science (Emtree), as shown in Figure 1.

**Figure 1:** Search strategy for the primary studies of the systematic review. Ribeirão Preto, SP, Brazil, 2019

Database	Controlled descriptors		Not controlled	Search strategy
**PubMed** **(MeSH)**	*Adult* *Young Adult* *Aged* *Qigong* *Treatment Outcome* *Outcome Assessment* *(Health Care)*		*Older Adults* *Qi Gong* *Ch’i Kung*	*(((((Adult) OR (Young Adult) OR (Older Adults) OR (Aged) OR (Elderly)))) AND (((Qigong) OR (Qi Gong) OR (Ch’i Kung)))) AND (((Treatment Outcome) OR (Outcome Assessment (Health Care))))*
**CINAHL** (MeSH)				
***Cochrane Central*** (MeSH)				
**EMBASE** (Emtree)	*Adult* *Young Adult* *Aged* *Qigong*	*Treatment Outcome* *Outcome Assessment* *(Health Care)*		
**LILACS** (DeCS)	*Adult* *Adulto* *Adulto* *Young Adult* *Adulto Joven* *Adulto Jovem* *Aged* *Anciano* *Idoso*	*Qigong* *Treatment Outcome* *Resultado del Tratamiento* *Resultado do tratamento* *Outcome Assessment (Health Care)* *Evaluación de Resultado (Atención de Salud)* *Avaliação de Resultado (Cuidados de Saúde)*		*(tw:((Adult) OR (Adulto) OR (Adulto) OR (Young Adult) OR (Adulto Joven) OR (Adulto Jovem) OR (Aged) OR (Anciano) OR (Idoso))) AND (tw:((Qigong) OR (Qi Gong) OR (Ch’i Kung))) AND (tw:((Outcome Assessment (Health Care)) OR (Evaluación de Resultado (Atención de Salud)) OR (Avaliação de Resultado (Cuidados de Saúde))))*

The following were adopted as inclusion criteria: randomized and non-randomized clinical trials related to the use of *Qigong* by adults and the elderly; which answer the research question; written in Portuguese, English or Spanish; beginning in January 2008 and ending in December 2018 (considering this as an important historical period for the recognition of Integrative and Complementary Practices [PICs] as the *Qigong* scientific and political circles); available on the mentioned bases. The exclusion criteria adopted were the following: not selecting research studies related to medical *Qigong* (external); which did not discuss assessing the effects of *Qigong* or did not present a correct definition of it; and which presented only clinical trial protocols, without obtaining results.

The search was carried out concomitantly in November 2018, through the five databases mentioned. When crossing the descriptors, 334 articles were found. In order to manage the research studies, the Rayyan QCRI^®^ and EndNote Web^®^ software were used. The same programs were used to export, organize, and filter studies with respect to duplication in the databases. The studies were pre-selected when reading titles and abstracts and, finally, they were selected for this review through complete reading in order to include them in the sample. Pre-selection was also carried out by reading the title and summary and, finally, selecting the studies for the final review by reading them in full for inclusion in the sample.

Data extraction was performed by two reviewers, independently. The recommendations of the Consolidated Standards of Reporting Trials^(^ 10 ^)^ were adopted for the careful reading of each included clinical trial, and a summary table was elaborated with the following information for the full analysis: identification (title and abstract); introduction (scientific basis, justification and objectives); method (type of study, inclusion and exclusion criteria of participants, interventions performed, hypotheses, sample size, randomization, blinding and statistical analysis); results (recruitment, baseline data, numbers analyzed, estimates, auxiliary analyzes and unwanted effects and damage in the groups analyzed); discussion (limitations, generalization and interpretation of results); conclusion; and protocol records. Both the research design and the level of evidence of the articles were carried out, so that it was possible to classify them according to the evidence hierarchy^(^ 11 ^)^.

The critical evaluation of the studies was carried out based on the recommendation of the Cochrane Collaboration^(^ 12 ^)^ for assessing the risk of clinical trial bias. It is a tool that allows for the assessment of several types of bias found in clinical trials, being organized into seven domains, with three categories each, namely: high risk of bias, low risk of bias, and uncertain risk of bias. Such an instrument was chosen because it was necessary to analyze the level of the research methodology found in the SR, so that the results from the scientific evidence in the literature were reliable.

## Results

**Figure 2:**
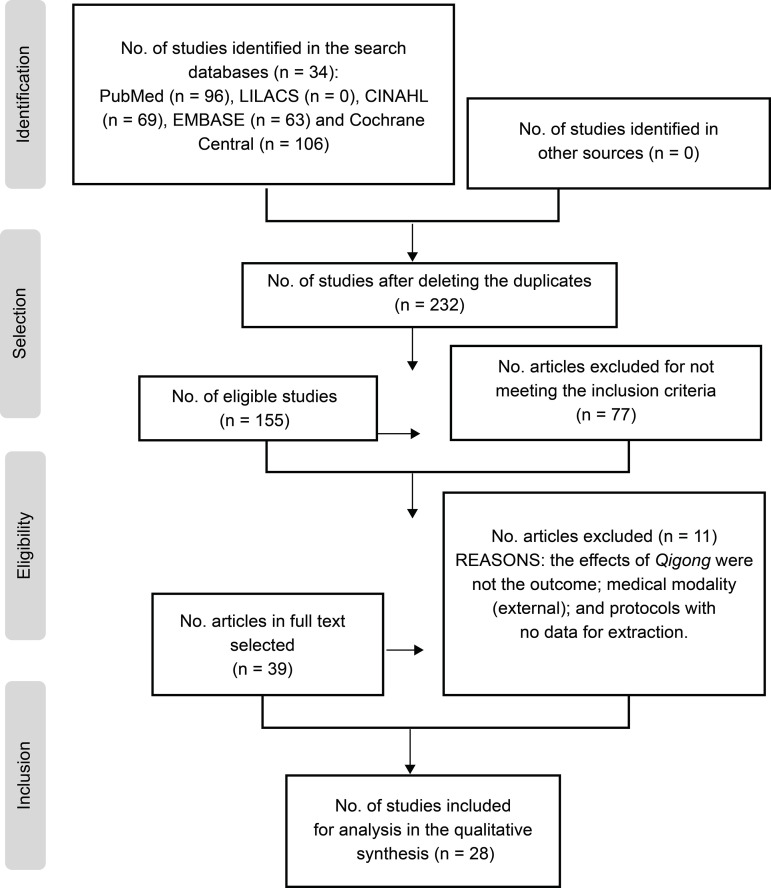
Flow of study selection, according to the adaptation of PRISMA^(^ 8 ^)^. Ribeirão Preto, SP, Brazil, 2019

28 studies were selected that were compatible with the inclusion criteria adopted for this SR, and studies were not added from the review of the references of this selected sample, as shown in Figure 2.

Regarding the main authorship of the articles, 13 (46.4%) were physicians, eight (28.6%) were nurses, two (7.1%) were psychologists, two (7.1%) were occupational therapists, one (3.6%) was a pharmacist, one (3.6%) was a physical educator, and one (3.6%) was a physical therapist. It was also noted that 15 (53.6%) of the studies were carried out in China, five (17.9%) in Germany, three (10.7%) in the United States, two (7.1%) in Sweden, two (7.1%) in Korea, and one (3.6%) in Thailand. Figure 3, presents the description of the studies analyzed in the SR.

**Figure 3:** Description of the studies included in the systematic review, according to year of publication and titles. Ribeirão Preto, SP, Brazil, 2019

Study	Year	Title
**E1**	2016	*Exploratory outcome assessment of Qigong/Tai Chi Easy on breast cancer survivor^(^13^)^.*
**E2**	2016	*Effect of health Baduanjin Qigong for mild to moderate Parkinson’s disease^(^14^)^.*
**E3**	2015	*Randomized controlled trial of Qigong/Tai Chi Easy on cancer-related fatigue in breast cancer^(^15^)^.*
**E4**	2016	*Qigong or Yoga Versus No Intervention in Older Adults With Chronic Low Back Pain: a Randomized Controlled Trial^(^16^)^.*
**E5**	2015	*Qigong versus exercise therapy for chronic low back pain in adults: a randomized controlled non-inferiority trial^(^17^)^.*
**E6**	2012	*Therapeutic Effects of Qigong in Patients with COPD: a Randomized Controlled Trial^(^18^)^.*
**E7**	2009	*Effects of Qigong in patients with burnout: a randomized controlled trial^(^19^)^.*
**E8**	2011	*Functional and Psychosocial Effects of Health Qigong in Patients with COPD: a Randomized Controlled Trial^(^20^)^.*
**E9**	2016	*Effects of Qigong Exercise on Biomarkers and Mental and Physical Health in Adults With at Least One Risk Factor for Coronary Artery Disease^(^21^)^.*
**E10**	2013	*Effects of a Brief Qigong-based Stress Reduction Program (BQSRP) in a distressed Korean population: a randomized trial^(^22^)^.*
**E11**	2013	*Psycho-physical and neurophysiological effects of qigong on depressed elders with chronic illness^(^23^)^.*
**E12**	2012	*A randomized controlled trial of qigong for fibromyalgia^(^24^)^.*
**E13**	2012	*A Randomized Controlled Trial of Qigong Exercise on Fatigue Symptoms, Functioning, and Telomerase Activity in Persons with Chronic Fatigue or Chronic Fatigue Syndrome^(^25^)^.*
**E14**	2011	*Tai chi Qigong improves lung functions and activity tolerance in COPD clients: a single blind, randomized controlled trial^(^26^)^.*
**E15**	2011	*Qigong Versus Exercise Versus No Therapy for Patients With Chronic Neck Pain: a randomized controlled trial^(^27^)^.*
**E16**	2010	*Qigong for the treatment of tinnitus: a prospective randomized controlled study^(^28^)^.*
**E17**	2009	*Qigong and exercise therapy for elderly patients with chronic neck pain (QIBANE): a randomized controlled study^(^29^)^.*
**E18**	2009	*Tai Chi Qigong for the quality of life of patients with knee osteoarthritis: a pilot, randomized, waiting list controlled trial^(^30^)^.*
**E19**	2008	*The effect of Qigong on Fibromyalgia (FMS): a controlled randomized study^(^31^)^.*
**E20**	2017	*Qigong/tai chi for sleep and fatigue in prostate cancer patients undergoing radiotherapy: a randomized controlled trial^(^32^)^.*
**E21**	2015	*The Effects of a 6-Month Tai Chi Qigong Training Program on Temporomandibular, Cervical, and Shoulder Joint Mobility and Sleep Problems in Nasopharyngeal Cancer Survivors^(^33^)^.*
**E22**	2016	*A randomized controlled trial of qigong on fatigue and sleep quality for non-Hodgkin’s lymphoma patients undergoing chemotherapy^(^34^)^.*
**E23**	2013	*The sustaining effects of Tai chi Qigong on physiological health for COPD patients: a randomized controlled trial^(^35^)^.*
**E24**	2017	*Effects of tai chi qigong on psychosocial well-being among hidden elderly, using elderly neighborhood volunteer approach: a pilot randomized controlled trial^(^36^)^.*
**E25**	2017	*A nurse facilitated mind-body interactive exercise (Chan-Chuang qigong) improves the health status of non-Hodgkin lymphoma patients receiving chemotherapy: Randomised controlled trial(37).*
**E26**	2017	*The efficacy of Guolin-Qigong on the body-mind health of Chinese women with breast cancer: a randomized controlled trial^(^38^)^.*
**E27**	2018	*Effects of Qigong practice in office workers with chronic non-specific low back pain: a randomized control trial^(^39^)^.*
**E28**	2014	*Effect of qigong training on fatigue in haemodialysis patients: a non-randomized controlled trial^(^40^)^.*

All the articles submitted for analysis were published in English, and 21 of them were in different journals with a predominance (n = 24) of medical journals. Only one was not randomized, although all were evaluated with level of evidence of II, considered strong, according to the theoretical framework adopted^(^ 11 ^)^.

**Figure 4:**
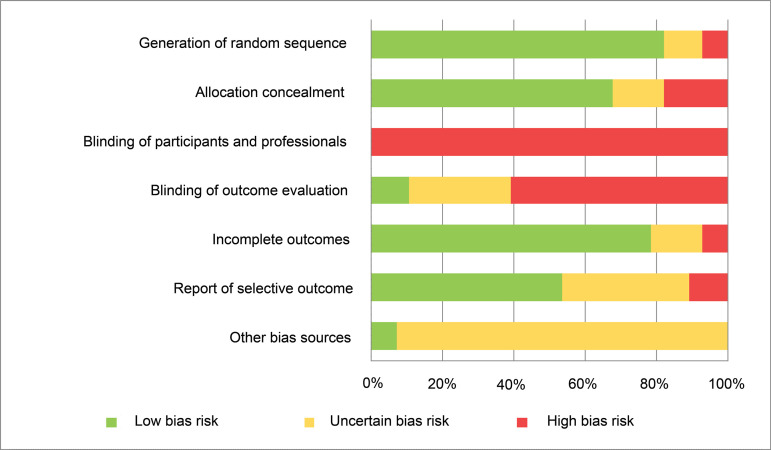
Bias risk assessment of the studies included in the systematic review, according to the Cochrane Collaboration assessment tool^(^ 12 ^)^. Ribeirão Preto, SP, Brazil, 2019

The works were critically evaluated, according to Figure 4, with the risk of bias assessment, in accordance with the criteria of the Cochrane Collaboration^(^ 12 ^)^ in order to determine the reliability of the results.

In order to fulfill the objective of this SR and to conduct a comparison between the data obtained and the literature, the articles were organized into five thematic categories. The first was established regarding health prevention associated with therapy, in which E9^(^ 21 ^)^, conducted with adults diagnosed with risk of Coronary Artery Disease (CAD) (n = 139) and with a mean age of 62 years old, showed an improvement in perceived mental and physical health (p < 0.001), and also in the amount of body fat (p < 0.001) compared to the control group. Also, the intervention (12 weeks) showed important results in lipid profiles, with significantly lower mean levels; however, not with regard to protein (hs-CRP), glycated hemoglobin (HbA1c), and fasting plasma sugar^(^ 21 ^)^.

The second thematic category dealt with the psychosocial effects caused by *Qigong* in three studies (E10, E11 and E24). E10^(^ 22 ^)^ held a *Qigong* program (4 weeks) in stressed subjects (n = 50) with a mean age of 38 years old showing that, compared to the control group, the intervention group had significantly greater reductions in the perceived stress scale scores (p = 0.0006), anxiety state (p = 0.0028), anxiety trait (p < 0.0001), personality subscales (p = 0.0321), symptoms (p = 0.0196) for rabies syndrome, as well as a significantly greater increase in the World Health Organization (WHO) QoL scores (p < 0.05). However, the salivary cortisol indexes did not change^(^ 22 ^)^.

E11^(^ 23 ^)^, study of *Qigong* (12 weeks) in depressed elderly people with chronic diseases (n = 38) and with a mean age of 80 years old, revealed that the intervention group showed a significant reduction in the depressive symptoms (p < 0.025), improved self-efficacy (p = 0.050), improved self-concept of physical well-being (p < 0.025), and increased physical strength (p = 0.034). In addition, a lower level of salivary cortisol (p < 0.008) was found in the referred group^(^ 23 ^)^.

E24^(^ 36 ^)^, conducted with socially isolated elderly individuals (n = 46) with an average age of 77 years old, showed that *Tai Chi/Qigong* (3 months) significantly improved the loneliness scale (p = 0.033), as well as the satisfaction component of the social support questionnaire (p = 0.044) when compared to the people in the control group. Another important fact is that 82% of the participants reported that they would continue the exercises, while nine reported that they extended their friendship circle, which indicates that the majority liked to practice *Tai Chi/Qigong* 36.

The third thematic category referred to the analgesic effects related to the practice of *Qigong*, in which five studies were analyzed (E4, E5, E15, E17 and E27). E4^(^ 16 ^)^, conducted with elderly people with low back pain (n = 176) with a mean age of 72 years old, showed no statistically significant distinctions in the groups regarding changes in the degree of low back pain during the survey ( *Qigong* x *Yoga*, p = 0.18, and *Qigong* x Control, p = 0.12). However, there was satisfaction with the *Qigong* therapy (7.9) and *Yoga* (7.8) and greater chances of recommending it to other individuals^(^ 16 ^)^.

E5^(^ 17 ^)^ investigated the therapy (3 months) in patients with chronic low back pain (n = 123) with a mean age of 46 years old, revealing that there was no statistical confirmation of non-inferiority (p = 0.204) - taking into account the margin of non-inferiority of 5 mm considered by the research in question in the mean intensity of low back pain - from the *Qigong* group (n = 64; 34.8 mm) when compared to the control group (n = 63; 33.1 mm), with the practice of stretching and relaxing for chronic low back pain. The distinction found was only the fact that the *Qigong* group adhered more in guided classes (67.2%), as well as those made at home (72%)^(^ 17 ^)^.

E15^(^ 27 ^)^ revealed that *Qigong* (6 months), in adults presenting chronic pain in the cervical region (n = 122) with a mean age of 45 years old, showed a significant difference with the control group with regard to the degree of pain (p = 0.002). The secondary results submitted to evaluation (neck pain, lack of capacity, and QoL) demonstrated the benefits of *Qigong* when compared to the control group, similarly when it comes to conventional exercises^(^ 27 ^)^. As for E17^(^ 29 ^)^, conducted with elderly people presenting chronic pain in the cervical region (n = 93) with a mean age of 75 years old, regarding pain, lack of capacity, and QoL, no significant distinctions were identified when comparing the *Qigong* (3 months) and control (p = 0.099) groups, as well as those with conventional exercise practices (p = 0.699)^29^.

Finally, E27^(^ 39 ^)^, conducted with adults reporting chronic and non-specific pain in the lower back and working in offices (n = 62) with a mean age of 35 years old, showed an important reduction in the degree of pain and functional disability in the lower back (p < 0.022 ) for people in the *Qigong* group (6 weeks). In addition, important improvements were identified with regard to the degree of pain (p < 0.001), functional impairment of the back region (p < 0.001), range of motion (p < 0.001), muscle strength (p < 0.001), heart rate (p < 0.001), and mental status (p = 0.005), in addition to better overall satisfaction with the practice (p < 0.001) in the intervention group^(^ 39 ^)^.

The fourth thematic category referred to the effects of the therapy in the area of oncology, in which seven studies (E1, E3, E20, E21, E22, E25 and E26) were grouped. E1^(^ 13 ^)^, conducted with 87 individuals who survived breast cancer, with a mean age of 59 years old, pointed to subsequent physical and psychological benefits (12 weeks), as well as an important increase in the level of physical activity and cognitive function (p < 0.001). There was also a reduction in body weight by the BMI [-0.66 (p = 0.048)] when compared to the other exercise group^(^ 13 ^)^.

E3^(^ 15 ^)^ tested an intervention (12 weeks) with Easy *Qigong/Tai Chi* versus *Sham Qigong* in relation to fatigue, depression, and sleep quality of people who survive breast cancer (n = 87) with a mean age of 58 years old. In this study, an important reduction in fatigue was found in the Easy *Qigong/Tai Chi* group (p = 0.005) compared to the other group, maintained throughout the 90 days of practice (p = 0.024). In addition, there was an improvement in depression and sleep in both interventions (p < 0.05)^(^ 15 ^)^.

As for E20^(^ 32 ^)^, which evaluated men with prostate cancer (n = 50) undergoing radiotherapy treatment with a mean age of 64 years old, it was noted that the group that practiced *Qigong* during the treatment described longer nights of sleep (p = 0.05) when compared to the control groups and to those who performed milder activities; however, at the end of radiotherapy, this no longer occurred^(^ 32 ^)^.

On the other hand, E21^(^ 33 ^)^, involving people who survived nasopharyngeal neoplasia (n = 52) and aged 58 years old, brought about positive results (6 months). The range of motion in the cervical region was improved (p < 0.008), and the mobility of the shoulders and temporomandibular joints did not change (p > 0.008). Worsening of the shoulder range of motion and of the ability to open the mouth was gradually identified in the control group (p < 0.008). Regarding sleep difficulties, there were improvements for the intervention group (p < 0.008), related to the development of the range of motion in the cervical region (p < 0.05)^(^ 33 ^)^.

E22^(^ 34 ^)^ assessed people affected by non-Hodgkin’s lymphoma undergoing chemotherapy (n = 102) with a mean age of 59 years old, also showing an important decrease in fatigue and an increase in sleep quality (p < 0.001) for *Qigong* (12 weeks), provided there is a considerable practice period^34^. In this sense, E25^(^ 37 ^)^, conducted with patients affected by the same disease (n = 96) with a mean age of 60 years old and submitted to the first cycle of chemotherapy treatment, also revealed an important improvement in the level of fatigue (p < 0.001) for the intervention group, in addition to white blood cells (p < 0.001), hemoglobin (p = 0.002), and sleep quality (p < 0.001)^(^ 37 ^)^.

In addition, E2^(^ 38 ^)^, conducted with participants rehabilitating from breast cancer (n = 158) with a mean age of 50 years old, there were benefits for emotional (p < 0.01) and specific (p < 0.01) well-being with regard to QoL submitted to evaluation and comparison with the control group for *Qigong* (24 weeks). There were benefits regarding anxiety (p < 0.01) for the intervention group. The control group had benefits for depression (p < 0.05). However, no significant distinction was identified between them. Both groups showed immunity benefits; however, in the *Qigong* one, it was more developed in the degrees of tumoral necrosis-alpha (TNF-a) (p < 0.05) compared to the control group^(^ 38 ^)^.

Finally, the fifth thematic category grouped twelve studies (E2, E6, E7, E8, E12, E13, E14, E16, E18, E19, E23 and E28) regarding the use of *Qigong* for health rehabilitation. E2^(^ 14 ^)^, conducted with patients with Parkinson’s Disease (n = 89), belonging to the 67-year-old age group, showed that the group practicing *Baduanjin* Qigong (6 months) presented important developments in sleep (p = 0.029), functional mobility (p = 0.041), and 6-minute walk test (p = 0.042) when compared to the control group. In addition, walking speed (p = 0.011) was increased^(^ 14 ^)^.

E6^(^ 18 ^)^, conducted with patients with Chronic Obstructive Pulmonary Disease (COPD) (n = 118) with a mean age of 62 years old, revealed that, even without being related to the stage of the disease, both the group that practiced *Qigong* conventional pulmonary rehabilitation activities improved with respect to the 6-minute walk test and QoL scores when compared to the control group. More precisely, those who practiced *Qigong* improved in decreasing acute exacerbation of COPD stage I, as well as the related complications, in addition to maintaining the stability of the TNF-a factor level for people with COPD stage II^(^ 18 ^)^.

As for E7^(^ 19 ^)^, conducted with participants with Burnout (n = 68) with a mean age of 44 years old, the results did not obtain statistically relevant distinctions in relation to the groups considered (12 weeks), since both had benefits in reducing the degrees of Burnout, fatigue, anxiety, and depression through *Qigong* ^(^ 19 ^)^.

Still with COPD participants (n = 52) with a mean age of 73 years, E8^(^ 20 ^)^ showed evidence for the development of functional capacity and QoL in all of its subscales in the group that practiced *Qigong*, while the other group showed signs of worsening QoL in four (general health, mental health, fatigue, and emotional)^(^ 20 ^)^.

Fibromyalgia was assessed in E12^(^ 24 ^)^(n = 89), in adults with a mean age of 52 years old, where the group that practiced *Qigong* (6 months) presented important pain-related benefits (2 months: p < 0.0001; 4 months: p = 0.0002; and 6 months: p = 0.02); impact of fibromyalgia (2 months: p < 0.0001; 4 months: p = 0.005; and 6 months: p = 0.02); sleep quality (2 months: p = 0.004; 4 months: p = 0.0007; and 6 months: p = 0.01); physical function (2 months: p = 0.001; 4 months: p = 0.009; and 6 months: p = 0.02); and mental function (2 months: p = 0.001; 4 months: p = 0.05; and 6 months: p = 0.35) in the QoL domains submitted to evaluation, compared to the control group^(^ 24 ^)^.

E13^(^ 25 ^)^, conducted with people showing chronic fatigue (n = 52) with a mean age of 42 years old, showed the effectiveness of the intervention (4 months) with important benefits compared to the control group regarding the total fatigue score (p < 0.05), physical fatigue (p < 0.01), mental fatigue (p < 0.05), and mental functioning (p = 0.001). Also, telomerase in the group with *Qigong* practitioners was statistically relevant compared to the control group (p < 0.05)^(^ 25 ^)^.

Regarding E14^(^ 26 ^)^, conducted with individuals with COPD (n = 206) with a mean age of 73 years old, improvements similar to other studies were found in the *Qigong* group (3 months) with regard to vital capacity (p = 0.002), forced expiratory volume in 1 second (p < 0.001), and exacerbation rate (p = 0.006). This was not evidenced in the conventional exercise group. Worsening of lung function was noted for the control group^(^ 26 ^)^.

Therapy was also assessed in E16^(^ 28 ^)^, conducted with individuals with tinnitus (n = 71) with a mean age of 45 years old, revealing that *Qigong* (5 weeks) improved tinnitus (p < 0.0001). Also, it is possible to consider that the intervention is potentially beneficial for the treatment of this condition given the great satisfaction of people with the therapy, as well as the stability of its effects (3 months)^(^ 28 ^)^. In E18^(^ 30 ^)^, conducted with people affected by symptomatic osteoarthritis with radiological changes in the knee joint (n = 44), with a mean age of 69 years old, the therapy showed statistically significant benefits for QoL in relation to the control group (p = 0.010), as well as in the 6-minute walk test (p = 0.005)^(^ 30 ^)^.

Individuals with Fibromyalgia Syndrome (FMS) (n = 57), with a mean age of 69 years old, were also studied in E19^(^ 31 ^)^, with an important decrease in pain (p < 0.0001), in addition to less inconvenience reported due to the condition (p < 0.0001) and better ability to keep it under control (p < 0.01) with therapy. Similarly, the group with people who practiced *Qigong* (7 weeks) reported a significant decrease in anxiety in relation to the control group (p < 0.01), in addition to a benefit in relation to QoL (p < 0.01)^(^ 31 ^)^.

Still regarding COPD, E23^(^ 35 ^)^, conducted with 206 participants with a mean age of 73 years old, showed an important development of exercise capacity (6 months) (p < 0.001), as well as a significant increase in the mean walking distance (+ 17%). A benefit for pulmonary functions was also identified (p < 0.001), as well as an improvement in the mean forced expiratory volume in 1 s (+ 11%)^(^ 35 ^)^.

Finally, E28^(^ 40 ^)^, conducted with patients undergoing hemodialysis (n = 172) with a mean age of 57 years old, revealed that, for the intervention group (6 months), fatigue was lower after 8 weeks of practice, with a significant decrease compared to the control group (p = 0.005). In addition, the research identified the benefit of *Qigong* for this audience in terms of strengthening muscles, developing psychological function, and reducing stress^(^ 40 ^)^.

## Discussion

The use of *Qigong* in the health network is revealed as a practice capable of promoting health, in addition to meeting prevention, which can be confirmed based on the positive results demonstrated in this study. That said, it is necessary to encourage studies focusing on the results of *Qigong*, considering prevention and health promotion, since most research studies seek to investigate people with established diseases.

Several research studies on the subject also indicated improvements in breathing, circulation, relaxation, and functions related to cognition due to the practice of *Qigong*, similarly to the findings of this study^(^ 41 ^-^ 43 ^)^. There was also evidence capable of positively associating the practice with the management of risk factors related to cardiovascular diseases, that is, *Qigong* is relevant to promote health and prevent illnesses for adults and for the elderly.

COPD patients, mostly elderly, also found benefits from the practice of *Qigong* regarding pulmonary function, functional capacity, quality of life, and reduced fatigue. Such results are in line with another SR on the relationship between *Qigong* and COPD^(^ 41 ^)^ with regard to the effects of therapy through breathing exercises techniques stimulated in the elderly, which projects great importance for planning the pulmonary rehabilitation of this population by the therapy.

Likewise, in this study, the practice had positive psychosocial results in elderly people, in line with related research studies, with similar results evidenced^(^ 44 ^-^ 46 ^)^. A number of studies on the improvement of depression in adults and the elderly by *Qigong* associate this result with the reduction of stress-related signals received by the limbic system, in the hippocampus and in the amygdala, in view of the connection between mind/attention with a confirmed focus through therapeutic practice, which consequently demonstrates having an effect on the secretion of the hormone responsible for releasing corticotrophin from the hypothalamus and, as well as from that adrenocorticotrophic (ACTH) by the hypophysis^(^ 47 ^-^ 48 ^)^.

As for analgesic effects, pain was one of the important outcomes investigated by the studies, with the literature showing that the therapy can enhance the level of tolerance and perception of pain, especially in adults. In this way, a relevant endogenous release of pain control is related to the practice of *Qigong* in the studies^(^ 49 ^-^ 50 ^)^. The effects of *Qigong* with regard to the pituitary-hypothalamus-adrenal axis, play an important role in the pathophysiology of fibromyalgia^(^ 49 ^,^ 51 ^)^, which makes it relevant to the recommendation and employment in the search for rehabilitating adults and the elderly.

As for Parkinson’s disease, studies in the area are in line with the results presented in this SR, proving that regular exercise practice, as proposed by *Qigong*, is beneficial for the rehabilitation of these patients, as it brings physical benefits, as well as a reduced chance that elderly people will suffer falls^(^ 52 ^)^.

With regard to the effects on oncology, a prospective longitudinal research has shown that it is even possible to consider the practice, inserted in the lifestyle, as protective with regard to the reduction of deaths due to neoplasms, by stimulating the immune system and controlling the inflammatory response related to the disease^(^ 53 ^)^.

It is also noted, in the research considered, an increase in the duration and intensity of the positive results of *Qigong*, proportional to the quantity practiced. This, therefore, needs to be considered to recommend engaging in research studies that aim to investigate the results of this practice for health. Therefore, an intervention protocol of at least four weeks is recommended, based on the results found in this SR.

*Qigong* is based on the development of self-knowledge, making its practitioner active and capable of preventing and curing diseases throughout life. Thus, *Qigong* is shown as a mind-body exercise modality that can be easily practiced by adults and very beneficial in the long term, as shown in the results of this study.

Through the critical evaluation conducted, it was possible to verify a high risk of bias in the research blinding domains, since this was impossible, and there was a probability of influencing the evaluation of the results, which lacked adequate control or, therefore, a clear description of the protocol and analysis^(^ 54 ^)^.

It is recommended to carry out new randomized clinical trials with a high level of methodology focusing on this research subject. In addition, in order to assess the practice of *Qigong*, longitudinal research studies involving healthy people are necessary to compose the baseline.

## Conclusion

It is possible to understand the physiological results from the practice of *Qigong* through the scientific evidence identified and submitted to evaluation, which makes this study of great contribution, since it brings a synthesis of the production regarding the use of an integrative and complementary practice in health, based on the most relevant scientific findings available.

The SR revealed the beneficial application of *Qigong* in the promotion, prevention, and rehabilitation of diseases and physiological disorders in adults and the elderly, such as: cancer; fibromyalgia; Parkinson’s disease; COPD; Burnout; stress; social isolation; chronic low back pain; cervical pain; buzz; osteoarthritis; fatigue; depression; and cardiovascular diseases. This reinforces the need to strengthen the use of PICs, such as the *Qigong*, in health care with a view to ensuring comprehensiveness and to improving the health care assistance offered to adults and elderly individuals.
